# Acquired Acrodermatitis Enteropathica in a 28-Year-Old Male with Type 1 Diabetes

**DOI:** 10.1155/2021/5572583

**Published:** 2021-05-25

**Authors:** Owen Ngalamika, Wencilaus M. P. Selvaraj, Fatima K. Yikona, Chibamba Mumba

**Affiliations:** ^1^Dermatology and Venereology Division, University Teaching Hospital, Lusaka, Zambia; ^2^University of Zambia School of Medicine, Lusaka, Zambia; ^3^Chelston Level One Hospital, Lusaka, Zambia; ^4^Pathology and Microbiology Department, University Teaching Hospital, Lusaka, Zambia

## Abstract

Acrodermatitis enteropathica (AE) is a rare disorder arising from inherited or acquired zinc deficiency. It is mainly characterized by acral dermatitis, periorificial dermatitis, alopecia, and gastrointestinal symptoms in the form of diarrhea. There are many complications of AE including local and systemic infections that may develop as a result of untreated AE. In addition, due to the role of zinc in glucose metabolism, chronic zinc deficiency may pose a challenge in the control of blood glucose levels in diabetics. We report the case of a 28-year-old male with type 1 diabetes who presented with signs and symptoms of AE.

## 1. Introduction

Acrodermatitis enteropathica (AE) is a rare disorder that is a result of a defect in zinc absorption. The primary form of AE is an autosomal recessive disorder due to mutations in the *SLC39A4* gene encoding a transmembrane zinc transporter protein [1]. The acquired form of AE may be due to inadequate zinc intake, conditions such as inflammatory bowel syndrome that may reduce zinc absorption, and excessive zinc excretion [2]. Classical clinical manifestations of AE include inflammation and/or blistering of the skin in the acral areas and around the mouth, hair loss, and diarrhea.

Zinc is an essential trace element that has many physiological functions including regulation of the immune system, wound healing, and growth [3, 4]. Zinc deficiency is associated with many conditions including diarrhea disease, lung and gastrointestinal infections, and poor wound healing [5, 6]. Here, we present the case of a young adult male who presented with signs and symptoms of AE.

## 2. Case Presentation

A 28-year-old male was referred from a primary-level hospital to the University Teaching Hospital (a tertiary-level hospital located in the city of Lusaka, Zambia) for adult onset of malnutrition. The patient had a history of generalized body swelling for 6 months. He also had loss of appetite and watery diarrhea (five times a day) for a duration of 5 months. The patient also gave a history of developing blisters in the lower limbs, upper limbs, and abdomen which later ruptured and peeled off resulting in hyperpigmentation of the affected areas. The lesions started on the lower limbs and then moved upwards to involve the trunk and upper limbs. He also gave a history of hair loss and discoloration. He denied other symptoms such as cough, fever, paroxysmal nocturnal dyspnea, or joint pains. Despite treatment at the local clinic, the condition continued to deteriorate, and therefore, he was referred to our tertiary institution for further management. The patient was a type 1 diabetic and was on insulin for 3 years prior to admission. He was said to be in good health before this illness. He gave a history of smoking and was a heavy alcohol drinker until he became unwell a few months before admission.

**Figure 1:**
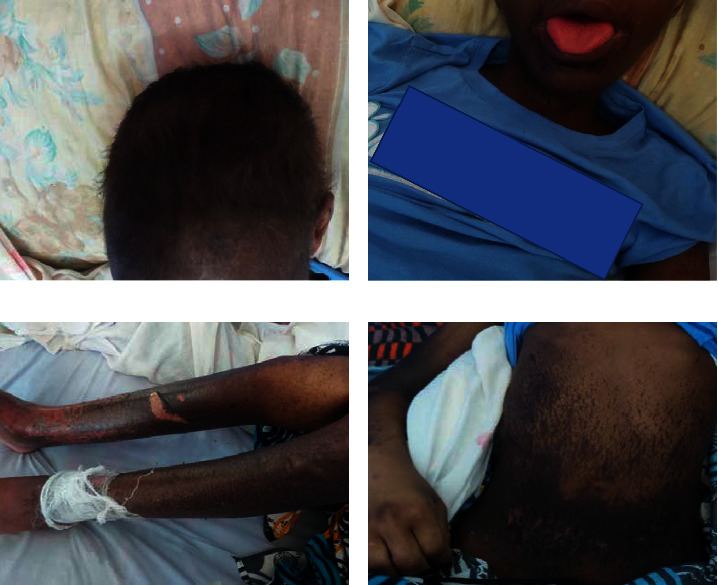
Dermatological manifestations on admission showing (a) hair dyspigmentation and alopecia; (b) perioral dermatitis and glossitis; and (c), (d) bullae and erosions.

On admission to the University Teaching Hospital, the patient underwent a full clinical assessment including laboratory investigations. On examination, the patient was lethargic and was unable to sit without support. His blood pressure was 93/60 mmHg, body temperature was 35.7°C, radial pulse rate was 89 beats per minute, respiratory rate was 24 breaths per minute, and peripheral oxygen saturation was 100%. He had conjunctival and palmar pallor with no signs of jaundice. He was noted to have generalized body swelling mostly involving the periorbital area, hands, and abdomen (ascites). His tongue was glossy and beefy red, and he had gingivitis and angular cheilitis. On local examination of the skin, he was noted to have hyperpigmented patches and erosions with some intact bullae on both the upper and lower limbs and the abdomen. The patient was also noted to have sparse hair with brownish discoloration (Figure 1). On examination of the genitalia, he was noted to have extensive skin erosions and scrotal swelling. Examination of the respiratory and cardiovascular systems was significant for a resting tachycardia and bibasal posterior crepitations. He had mild pedal edema on the lower limbs, feet, and hands. In addition, he had an infected ulcer on the left antecubital fossa.

**Figure 2:**
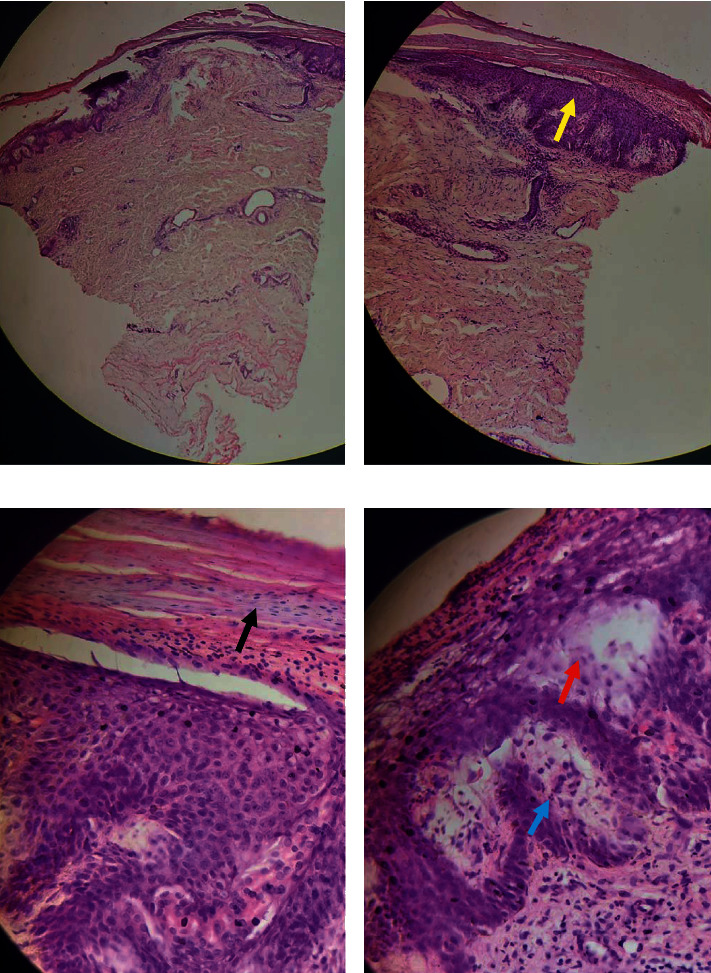
Hematoxylin-and-eosin-stained skin biopsy showing (a) the epidermis and dermis; (b) acanthosis (yellow arrow); (c) parakeratosis (black arrow); and (d) neutrophilic epithelial infiltrate (blue arrow) and spongiosis (red arrow). (a) ×100, (b) ×200, and (c) and (d) ×400 magnification.

**Figure 3:**
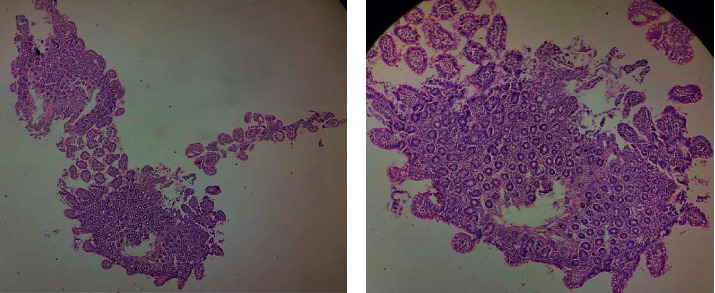
Hematoxylin-and-eosin-stained ileal biopsy showing mild focal loss and blunting of the villi.

A number of laboratory investigations were conducted which included a complete blood count which showed severe anemia, liver function test which revealed a hypoalbuminemia, and kidney function tests which were normal (Table 1). A pus swab for microscopy and culture collected from the infected ulcer located on the left antecubital fossa showed the presence of *Staphylococcus aureus* and Acinetobacter species. Blood culture initially showed *Staphylococcus aureus*, and a repeat blood culture performed two weeks later showed the presence of yeast cells and Gram-negative bacilli. Other organisms identified on the same culture were *Candida* and *Klebsiella pneumoniae* species. A skin biopsy of an intact lesion was performed, and it showed features consistent with AE including spongiosis with epidermal pallor, parakeratosis, and keratinocyte necrolysis (Figure 2). Upper gastrointestinal endoscopy was performed and showed villous blunting, and biopsies of the duodenum and ilium were also performed and showed blunting of the villi (Figure 3).

**Figure 4:**
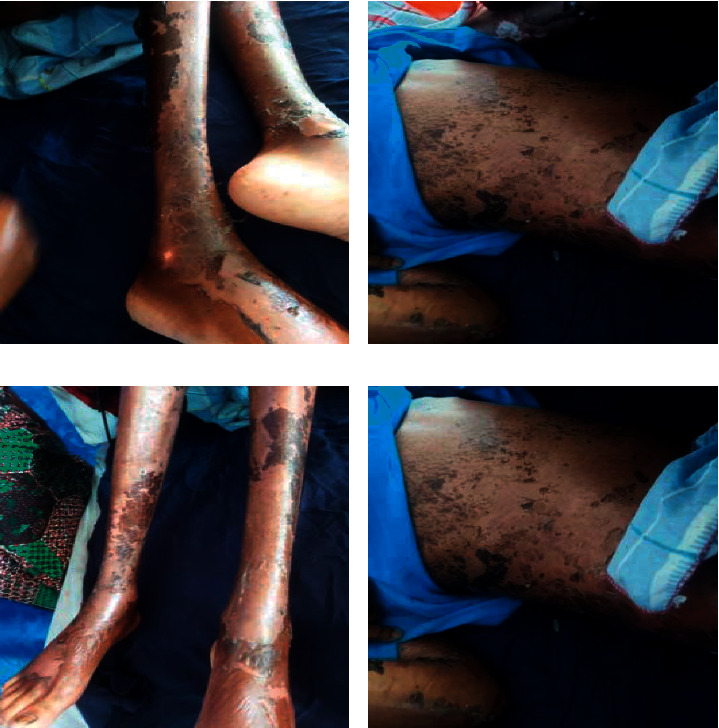
Skin changes after zinc replacement with evident re-epithelialization after about a week of zinc supplementation.

A diagnosis of acquired AE with sepsis was made based on the clinical findings. The differential diagnosis was pemphigus foliaceous. The patient was initiated on oral zinc sulphate at 50 mg three times a day with hematinics (folic acid and ferrous sulphate) and antibiotics. A few days after initiation of zinc, the patient showed remarkable improvement in the skin lesions including a reduction in the edema, resolution of diarrhea, and improvement in appetite (Figure 4). Measurement of plasma zinc levels was performed several days after the patient had been commenced on zinc sulphate and showed high zinc levels. On admission, he also received a blood transfusion for the anemia.

Despite improvement in the skin lesions and diarrhea, the patient's general condition continued to deteriorate. His sugar levels were fluctuating with episodes of hyper- and hypoglycemia, and he continued to have signs and symptoms of sepsis. The patient eventually died after 25 days of admission.

## 3. Discussion

AE is a rare disorder resulting from zinc deficiency. Primary AE is commonly diagnosed in childhood and has a strong genetic basis [7]. Acquired AE on the other hand is commonly a secondary manifestation of malabsorption syndromes [8]. While it is relatively easy to make a diagnosis of primary AE, secondary AE usually requires an exclusion of other possible disorders and also diagnosing other conditions that may predispose to zinc deficiency. Laboratory diagnostic tests for acquired AE are neither sensitive nor specific, and therefore, clinicians mostly depend on how the patient responds to zinc therapy [9].

Our patient had classical signs and symptoms of AE including perioral dermatitis, bullous eruptions in the acral areas and extremities, alopecia, and diarrhea. In addition, he also had other signs that were highly suggestive of AE including glossitis, anemia, and infections. His age at initial presentation was suggestive of the acquired instead of the primary form of AE. In addition, his history of heavy alcohol intake also supported a diagnosis of acquired AE as previously reported [8].

Individuals with zinc deficiency are susceptible to inflammatory dermatoses, which may include bullous eruptions, perioral dermatitis, and glossitis, as seen in our patient. Zinc is important in keratinocyte proliferation and differentiation [10]. Thus, zinc deficiency results in keratinocyte apoptosis [11] and poor differentiation [12], which were evident in our patient both clinically (bullae) and on pathology (parakeratosis).

Our patient had a skin infection and sepsis. Zinc is important in both innate and adaptive immune responses [13]. Zinc deficiency can result in an impaired innate immune system including reduced natural killer cell activity, impaired chemotaxis, phagocytosis, and microbicidal activity of neutrophils and monocytes/macrophages [5]. Zinc deficiency can also lead to a defective adaptive immune system by impairing the development and function of T cells [14]. Low levels of zinc in plasma are associated with an increased incidence of infections in the elderly [15]. Persistently low levels of serum zinc have also been associated with recurrent sepsis in critically ill patients [16]. In addition, zinc oxide nanoparticles have been observed to have antibacterial activity against infections by multiple organisms including methicillin-resistant *Staphylococcus aureus* [17]. Zinc deficiency may have been the predominant predisposing factor to the cutaneous infection and sepsis observed in our patient.

Our patient also had diarrhea, a common feature in AE. Zinc plays a key role in promoting growth and differentiation of enterocytes and inhibiting the excessive secretion of fluids and ions caused by some organisms including *Vibrio cholerae* and *Shigella* [18]. In addition, zinc is important in development and function of the innate and adaptive immune responses [18]. In particular, zinc has been shown to enhance innate immunity against enterotoxigenic *Escherichia coli* [19]. Both the skin and gastrointestinal infections could have been responsible for the sepsis observed in our patient, and zinc deficiency was most likely a significant predisposing factor for these infections.

Our patient was also a type 1 diabetic. Low serum zinc levels have been associated with poor glycemic control in both type 1 and 2 diabetics [20, 21]. Zinc is a cofactor in the synthesis, storage, and secretion of insulin [20]. In addition, since zinc is essential in the formation and function of antioxidant enzymes, zinc deficiency may result in an increase in intracellular oxidants and free radicals in diabetics [22]. The poor control of blood sugar levels in our patient could be attributed to the chronic hypozincemia.

## 4. Conclusions

Zinc deficiency in the form of AE is an uncommon condition that may lead to life-threatening complications if left untreated. Clinicians should have a high index of suspicion for possible zinc deficiency so as to treat the condition early and prevent the complications that may arise from it.

## Figures and Tables

**Table 1:** Laboratory investigations conducted.

	On admission	After admission	Reference range
Complete blood count^∗^			
White cell count (×10^9^/l)	6.30	7.20	4.00–10.00
Hemoglobin (g/dl)	6.5	8.5	13.0–17.00
Mean cell volume (fl)	82.3	82.9	83.0–101.0
Mean cell hemoglobin (pg)	26.7	25.7	27.0–32.0
Platelet count (×10^9^/l)	155	123	150–410

Liver function tests			
Albumin (g/l)	10.6		35.0–52.0
Total protein (g/l)	36.6		60.0–78.0
Serum calcium (mg/dl)	1.48		2.15–2.50
Bilirubin (*μ*mol/l)	5.4		2.0–21.0
Alanine transaminase (u/l)	37.7		0.0–45.0
Aspartate transaminase (u/l)	23.1		0.0–35.0

Kidney function tests			
Urea (mmol/l)	1.74		2.80–7.10
Creatinine (*μ*mol/l)	55.9		59.0–104.0

Other tests			
Random blood sugar^∗^	4.8	9.4	<11.0
Serum zinc (*μ*mol/l)^#^	Unknown	22.4	10.7–18.4

^∗^Repeat complete blood count and random blood sugar determined 2 days after admission; ^#^serum zinc determined after 11 days of admission.
